# Late-effect awareness and follow-up of cancer in general practice

**DOI:** 10.1080/02813432.2022.2139457

**Published:** 2022-11-15

**Authors:** Siri A. Eikeland, Knut B. Smeland, Mette Brekke, Cecilie E. Kiserud, Alexander Fosså

**Affiliations:** a National Advisory Unit for Late Effects after Cancer Treatment, Department of Oncology, Oslo University Hospital, Oslo, Norway; b Institute of Clinical Medicine, University of Oslo, Oslo, Norway; c General Practice Research Unit, Institute of Health and Society, University of Oslo, Oslo, Norway; d Department of Oncology and Radiotherapy, Oslo University Hospital, Oslo, Norway

**Keywords:** Late effects; Hodgkin's lymphoma; General practice; Survivorship care

## Abstract

**Objective:**

With increasing cancer incidence and survival rates, follow-up care becomes a major healthcare concern, placing increased demands on general practitioners (GPs). We explored GPs’ awareness of late effects (LEs) after cancer treatment. Their degree of involvement and attitudes towards follow-up care was studied separately for solid cancers and Hodgkin’s lymphoma (HL).

**Design and setting:**

Mailed questionnaire study in Norwegian general practice.

**Subjects:**

185 responding GPs with responsibility for HL survivors, more than 10 years since diagnosis.

**Main outcome measures and results:**

All GPs reported some awareness of LEs. Increasing awareness of LEs was associated with female sex, being a specialist, having experience from hospital-based cancer care and familiarity with official guidelines on LEs after treatment. The majority of GPs were involved in follow-up care, which increased with patients’ time since treatment and was associated with higher awareness of LEs. GPs with work experience in hospital-based cancer care were more likely to be engaged in HL follow-up. Most GPs were willing to provide follow-up care at some point after treatment. Older and more experienced GPs, and those satisfied with the collaboration with hospital specialists, were more likely to provide follow-up earlier.

**Conclusion:**

GPs’ awareness of LEs and their willingness to provide follow-up care were related to familiarity with guidelines and experience. GPs more involved in follow-up care also had higher knowledge of LEs. Distribution of guidelines on LEs and follow-up care, and improving collaboration with hospital specialists, might increase GPs’ knowledge and willingness to become involved in follow-up care, especially early in their careers. GPs’ involvement and attitude towards follow-up of survivors of common solid cancers and HL, a rare malignant disease, were similar.

**KEY POINTS**
Norwegian general practitioners (GPs) are involved in survivorship care after cancer treatment. We investigated their awareness of late effects (LEs), their involvement and their attitude towards follow-up care of solid cancers and Hodgkin’s lymphoma. • GPs registered as specialists, aware of guidelines and with experience from hospital-based cancer care reported higher awareness of LEs.
  • GPs with higher awareness of LEs were more frequently involved in follow-up care.
  • GPs with longer experience in general practice were comfortable with follow-up care at an earlier stage after treatment.
  • Results were similar for follow-up care of survivors of solid cancers and Hodgkin’s lymphoma.

## Introduction

As cancer diagnostics and treatment improve, an increasing number of patients survive, and place higher demands on healthcare services. According to the Norwegian Cancer Registry, in 2020, the relative 5-year survival of all cancer patients was about 77% [1]. At the end of 2020 more than 300,000 Norwegians were alive after a diagnosis of cancer, the highest prevalence was seen for prostate cancer (57,000), breast cancer (53,000), melanoma of the skin (30,000) and colon cancer (25,000). About 3000 were Hodgkin’s lymphoma (HL) survivors [1].

Following treatment, cancer survivors in Norway are normally followed-up by hospital specialists for a varying period of time, before being transferred to their general practitioner (GP) for subsequent care. Important goals of survivorship care involve detecting relapse and/or late effects (LEs) after treatment, assisting with rehabilitation and access to social benefits, and follow-up of comorbidities together with other health issues. Determining GPs’ role in follow-up care [2] and enabling them to take on this responsibility are therefore national prioritized goals [3].

LEs after cancer can be defined as adverse health outcomes or complications of the disease itself or the treatment, lasting for more than a year, or occurring more than a year after treatment completion [4]. They can be of somatic, psychological or social character, and as such affect both quality of life and survival. Awareness concerning LEs appears to be a cornerstone in cancer survivorship care, both within specialist- and primary healthcare systems. The Norwegian Directorate of Health has published guidelines concerning LEs after cancer treatment in general [5] and radiotherapy for lymphoma[6], in addition to guidelines issued on both treatment and follow-up care for various cancer diagnoses, for example, colorectal [7], breast [8], prostate [9] and lymphomas [10].

Compared to most solid cancers, HL is a rare malignant disease affecting relatively young patients [11], most of whom will become long-time survivors in need of follow-up for decades after therapy [12]. Historically, HL survivors have been at a particularly high risk of LEs, mainly due to the frequent use of radiotherapy and combination chemotherapy, including potentially severe complications such as heart disease and secondary cancers [13]. Further, there may be suboptimal delivery of survivorship information, important for the quality of follow-up, especially when survivorship care is transferred to primary care providers [14]. Being a relatively small patient group [1] few GPs will however acquire a lot of experience providing follow-up care for HL survivors, compared to survivors of more prevalent solid cancers, possibly affecting their attitude towards such care.

We aimed to study Norwegian GPs’ awareness of LEs after cancer treatment in general. Further, we wanted to study their involvement in and attitudes towards follow-up care of survivors of common solid cancers and HL lymphoma separately, as the latter is a relatively infrequent, young and possibly complex group of survivors. Therefore, HL survivors might be perceived differently by GPs, to our knowledge a comparison not explored in previous studies.

## Material and methods

### Study population

GPs were identified, on the basis of a national study from 2017/2018, of HL survivors treated between 1997 and 2006, where 301 participants had given written consent to contact their respective GP [15]. Two survivors had died and one did not have a registered GP, resulting in 298 eligible GPs contacted with a mailed questionnaire during 2020. Non-respondents received one written reminder. The GPs were from three health regions; South-East, Mid and North of Norway.

### Questionnaire

We used a 26-item questionnaire (Table 1), modified from a 13-item questionnaire developed by Fidjeland et al. on GPs’ experience with and attitudes towards cancer follow-up care [16]. In addition, we included items exploring GPs’ awareness of LEs after cancer treatment in general, their involvement and their attitude towards follow-up care of HL survivors. The questionnaire was divided into four sections:

**Table 1.** Characteristics of the general practitioners.

	*n* (%)^b^
GP survey invitations	298
Responders, *n* (%)	185 (62)
Sex, *n* (%)	
Male	99 (54)
Female	86 (46)
Age, years, mean (SD)	48 (12)
Registered specialist, *n* (%)^a^	125 (68)
Experience, years, mean (SD)^a^	17 (12)
Experience with hospital-based cancer care, *n* (%)	
Yes	63 (34)
No	120 (66)
Awareness of LEs after cancer treatment	
Perceived awareness of LEs after cancer treatment, *n* (%)	
None	0
Some	113 (61)
A lot	72 (39)
Source of information	
Medical practice/experience	150 (81)
Specialization in general practice	89 (48)
University studies/medical education	85 (46)
Official information	58 (31)
Self-studies	33 (18)
Other	21 (11
Awareness about LEs after HL therapy, *n* (%)	171 (97)
Awareness on LEs after cancer treatment in general, *n* (%)	
Fatigue	170 (92)
Peripheral neuropathy	133 (72)
Cardiovascular disease	129 (70)
Reduced fertility	94 (51)
Hormonal disturbances	78 (42)
Other^c^	34 (18)
Late effect awareness score, mean (SD)	3.45 (1.46)
Awareness of guidelines on LEs after cancer treatment, *n* (%)	
Yes	46 (25)
In use by the GP	34 (74)
GP’s perceived degree of usefulness	
Very	12 (26)
To some degree	24 (52)
Not at all	1 (2)
Awareness of guidelines on LEs after radiotherapy for lymphoma, *n* (%)	
Yes	23 (12)
In use by the GP	8 (35)
GP’s perceived degree of usefulness	
Very	3 (13)
To some degree	9 (39)
Not at all	0
Involvement in follow-up care for survivors	
<5 years since treatment, *n* (%)	
Breast cancer	
Never	33 (18)
Rarely	84 (45)
Frequently	66 (36)
Colorectal cancer	
Never	21 (11)
Rarely	102 (55)
Frequently	61 (33)
Prostate cancer	
Never	19 (10)
Rarely	73 (40)
Frequently	92 (50)
Other cancer	
Never	20 (13)
Rarely	85 (57)
Frequently	44 (30)
Hodgkin’s lymphoma	
Never	61 (34)
Rarely	116 (64)
Frequently	3 (2)
Collaboration with hospital, *n* (%)	149 (87)
≥5 years since treatment, *n* (%)	
Breast cancer	
Never	13 (7)
Rarely	64 (35)
Frequently	107 (58)
Colorectal cancer	
Never	12 (7)
Rarely	76 (42)
Frequently	94 (51)
Prostate cancer	
Never	8 (4)
Rarely	57 (31)
Frequently	119 (65)
Other cancer	
Never	13 (9)
Rarely	70 (49)
Frequently	60 (42)
Hodgkin’s lymphoma	
Never	27 (15)
Rarely	143 (79)
Frequently	10 (6)
Collaboration with hospital, *n* (%)	82 (47)
Attitude towards follow-up care	
Perceived important responsibility for GP in follow-up, *n* (%)	
Detect relapse	166 (90)
Assist other health related issues	134 (72)
Assist rehabilitation	128 (69)
Assist sick leave, disability pension and social welfare	112 (61)
Provide information about Les	81 (44)
Opinion on collaboration with hospital, *n* (%)	
Very good	25 (14)
Good	69 (38)
Acceptable	69 (38)
Poor	19 (10)
Very poor	2 (1)
Perceived challenges in follow-up care, *n* (%)	
Uncertainty about role and division of responsibilities	118 (64)
Uncertainty about guidelines for follow-up care	98 (53)
Deficient and/or delayed discharge summaries	62 (34)
Unavailable specialist appointments	49 (27)
Time point after treatment where GPs feel comfortable with responsibility for follow-up after solid cancer, *n* (%)	
Immediately, 0–1 years	10 (6)
>1–3 years	60 (33)
>3–5 years	56 (31)
>5 years	50 (27)
Never	7 (4)
Obstacles taking responsibility for follow up after solid cancer treatment at any time point, *n* (%)	
Lack of interest	0
GP’s inadequate professional competence	4 (57)
Time constraints/work load	4 (57)
Hospital specialists’ higher level of competence	5 (71)
Time point after treatment where GPs feel comfortable with responsibility for follow-up after lymphoma, *n* (%)	
Immediately, 0–1 years	9 (5)
>1–3 years	52 (28)
>3–5 years	51 (28)
>5 years	62 (34)
Never	9 (5)
Obstacles taking responsibility for follow up after lymphoma treatment at any time point, *n* (%)	
Lack of interest	0
GP’s inadequate professional competence	4 (44)
Time constraints/work load	4 (44)
Hospital specialists’ higher level of competence	7 (78)

^a^Experience and specialization in general practice.

^b^Percentages based on number of valid responses, missing responses in between 1 and 42.

^c^Other LEs including 16 (9%) responding on secondary malignancies.

GP: general practitioner; HL: Hodgkin’s lymphoma; LEs: late effects.

- Sex, age, specialist status, years of experience in general practice and experience from hospital-based cancer care.
- Awareness of LEs and recommendations on follow-up care. GPs were asked whether they were aware of 5 specified LEs (infertility, cardiovascular disease, peripheral neuropathy, fatigue, hormonal disturbances), in addition to the option of stating awareness of one ‘other relevant LEs’. By summing up positive responses, we generated a LE awareness score (ranging from 0-6) for each GP. The GPs were also asked about familiarity and opinion on publications from the Norwegian Directorate of Health on LEs and follow-up care after cancer treatment in general [5] and follow-up care and prevention of LEs after radiotherapy for lymphoma [6], respectively.
- Annual frequency of contact (frequent: ≥5 patients, rare: <5 patients, or no contact) with survivors after common solid cancers and HL, structured by time since the end of treatment (<5 years and ≥5 years). GPs were grouped according to the number of cancer diagnoses for which they provided frequent follow-up care, ‘0’ (i.e. no solid cancer with ≥5 survivors per year), ‘1–2’ (i.e. one or two diagnoses with ≥5 survivors per year) and ‘3–4’ (similarly 3 or 4 diagnoses with ≥5 survivors per year), both for survivors within and beyond 5 years since treatment. Very few GPs had frequent contact with HL survivors, and involvement in follow-up was dichotomized into ‘frequently/rarely’ or ‘never’ both within and beyond 5 years since treatment.
- Attitudes towards follow-up care after treatment of solid cancer and HL. Questions addressed the time point since treatment where GPs feel comfortable taking responsibility for follow-up (0–3, >3–5 and >5 years since treatment or never), and GPs’ experience, perceived roles and challenges in both follow-up care and collaboration with hospital specialists.

### Statistics and ethics

Categorical data were presented as absolute numbers and percentages, and continuous data as means and standard deviations (SD). Groups of survivors were compared using Chi-square test, two-sample *t*-test and ANOVA as appropriate. Uni- and multivariate linear regression analyses evaluated factors associated with GPs’ awareness of LEs. All tests were two-sided, and *p*-values <0.05 were considered statistically significant. Data analysis was performed using Statistical Package for the Social Sciences (SPSS).

The Regional committee for medical and health research ethics South East approved the study (2016/2311).

## Results

### Demographics

Of 298 invited GPs, 185 (62%) responded to the questionnaire (Table 1). The mean age was 48 years, 54% were men, 68% were specialists in general practice and a mean of 17 years of experience in general practice. The majority had no experience with hospital-based cancer care (66%). The study sample was representative of all registered GPs in Norway in terms of age (mean 47 years), sex (54% men) and proportion of registered specialists in general practice (63%) [17].

### Awareness of LEs after cancer treatment

Of the responding GPs, all reported to have some degree of awareness of LEs after cancer treatment in general, and most were familiar with the risk of LEs after HL treatment in particular (Table 1). The main sources for awareness on LEs were medical practice, specialization in general practice and/or university education. Guidelines on LEs after cancer therapy in general [5] or radiotherapy after lymphoma [6] were known to 25% and 12% of the GPs respectively, but if known, generally stated to be in use and found to be useful. The majority recognized fatigue, cardiovascular disease and peripheral neuropathy as common LEs and about one-half recognized the risk of reduced fertility and hormonal disturbances (Table 1). Other LEs, not included in the 5 pre-specified conditions, were recognized by 34 GPs, of whom 16 stated secondary cancer.

The mean LE awareness score was 3.45 (SD = 1.46) (Table 1). In linear regression analysis, significant univariate associations with the LE awareness score were found for being a specialist in general practice and familiarity with both aforementioned guidelines on LEs [5,6] (Table 2). In multivariate analysis, including as independent predictors GPs’ sex and all variables with a *p*-value below 0.2 in univariate tests (age excluded due to collinearity with experience in general practice), the LE awareness score was significantly associated with being female, a registered specialist, familiarity with guidelines on LE after cancer treatment in general, as well as having experience from hospital-based cancer care (Table 2).

**Table 2.** Factors associated with general practitioners’ awareness of late effects.

	Late effect awareness score^a^					
	Univariate			Multivariate		
Characteristic	B^c^	*p* ^d^	95% CI^e^	B^c^	*p* ^d^	95% CI^e^
Sex (female vs male)	0.23	0.29	−0.20 to 0.65	0.46	**0.03**	0.05–0.86
Age (years)	0.02	0.07	−0.01 to 0.03			
Experience (years)^b^	0.02	0.06	−0.001 to 0.03	0.001	0.90	−0.02–0.02
Experience with hospital-based cancer care (yes vs no)	0.29	0.19	−0.15 to 0.74	0.49	**0.02**	0.07–0.92
Registered specialist (yes vs no)^b^	0.71	**0.002**	0.25–1.16	0.61	**0.02**	0.11–1.11
Awareness of guidelines on LEs after cancer treatment (yes vs no)	0.97	**<0.001**	0.50–1.43	0.85	**0.001**	0.35–1.35
Awareness of guidelines on LEs after radiotherapy for lymphoma (yes vs no)	0.93	**0.004**	0.30–1.56	0.52	0.13	−0.15−1.19

^a^Linear regression analysis with late effect awareness score as the dependent variable, that is, number of late effects (infertility, cardiovascular disease, peripheral neuropathy, fatigue, hormonal disturbances or ‘other’) of which the respondent reported awareness.

^b^Experience and specialization in general practice.

^c^B, unstandardized regression coefficient.

^d^*p*-Values below 0.05 in bold.

^e^CI: confidence interval for unstandardized coefficient.

LEs: late effects.

### Involvement in cancer and lymphoma follow-up care, and associated factors

Between 30% - 50% of GPs reported to provide follow-up care ‘frequently’ to survivors of breast, colorectal-, prostate- and other solid cancers within 5 years since treatment, with an increase to 42–65% beyond 5 years (Table 1). Grouping GPs according to the number of solid cancer diagnoses for which they saw ≥5 survivors per year (0, 1–2 or 3–4), we determined characteristics of GPs providing care for survivors of solid cancers at varying frequencies (Table 3). GPs with more frequent involvement in solid tumor follow-up had higher LE awareness scores, both for survivors within (*p* = 0.008) and beyond (*p* = 0.002) the first 5 years since treatment, but did not differ significantly in terms of other characteristics.

**Table 3.** General practitioners’ level of involvement in follow up after solid cancer and Hodgkin’s lymphoma.

	Number of solid cancer diagnoses with frequent follow-up^a,b^				Involvement in HL follow-up^b^		
	3-4	1-2	0	*p^c^*	Involved^d^	Not involved	*p^c^*
	<5 years since treatment						
Characteristic							
*n* (%)	55 (30)	60 (32)	70 (38)		119 (66)	61 (34)	
Age, years, mean (SD)	50 (12.1)	48.9 (12.1)	45.8 (12.5)	0.14	48.8 (12.2)	47.2 (12.8)	0.41
Experience, years, mean (SD)^e^	18.5 (12.7)	17.8 (11.6)	14.4 (12.3)	0.12	17.8 (12.6)	15.2 (11.6)	0.18
Registered specialist, *n* (%)^e^	44 (80)	40 (69)	41 (60)	0.06	82 (70)	40 (68)	0.82
Sex, male, *n* (%)	31 (56)	36 (60)	32 (46)	0.23	68 (57)	28 (46)	0.15
Experience hospital-based cancer care, *n* (%)	20 (36)	23 (40)	20 (29)	0.40	48 (41)	15 (25)	**0.04**
LE awareness score, mean (SD)	3.6 (1.5)	3.8 (1.3)	3.0 (1.5)	**0.008**	3.5 (1.5)	3.4 (1.4)	0.53
Awareness of guidelines on LEs after cancer treatment in general, *n* (%)	18 (33)	15 (25)	13 (19)	0.19	34 (29)	12 (20)	0.20
Awareness of guidelines on LEs after radiotherapy for lymphoma, *n* (%)	9 (16)	7 (12)	7 (10)	0.55	18 (15)	5 (8)	0.19
	≥5 years since treatment						
*n* (%)	93 (50)	41 (22)	51 (28)		153 (85)	27 (15)	
Age, years, mean (SD)	48.3 (12.1)	50.4 (12.7)	45.7 (12.2)	0.18	48.3 (12.5)	46.4 (11.4)	0.43
Experience, years, mean (SD)	17.1 (12.3)	18.9 (12.4)	14.3 (11.9)	0.18	16.9 (12.4)	14.8 (10.4)	0.36
Registered specialist, *n* (%)	66 (72)	30 (75)	29 (59)	0.20	106 (70)	17 (68)	0.86
Sex – male, *n* (%)	47 (51)	23 (56)	29 (57)	0.72	82 (54)	13 (48)	0.60
Experience with hospital-based cancer care, *n* (%)	29 (31)	19 (46)	15 (31)	0.19	59 (39)	4 (15)	**0.02**
LE awareness score, mean (SD)	3.8 (1.4)	3.4 (1.4)	2.9 (1.5)	**0.002**	3.6 (1.5)	2.6 (1.3)	**0.001**
Awareness of guidelines on LEs after cancer treatment in general, *n* (%)	28 (30)	8 (20)	10 (20)	0.25	40 (26)	5 (19)	0.40
Awareness of guidelines on LEs after radiotherapy for lymphoma, *n* (%)	13 (14)	4 (10)	6 (12)	0.78	21 (14)	2 (7)	0.37

Frequency measure: number of solid cancer diagnoses (breast, colorectal, prostate and ‘other’) for which the GP reported to be involved in follow-up care at a frequent basis (>5 survivors per year).

^b^Percentages based on number of valid responses, missing responses in between 2 and 8.

^c^*p*-Value for Chi-square test for categorical variables and *t*-test/ANOVA for continuous variables. *p*-Values below 0.05 in bold.

^d^Frequently and rarely combined, only 3 answered ‘frequently’.

^e^Experience and specialization in general practice.

HL: Hodgkin’s lymphoma; SD: standard deviation; LEs: late effects.

Regarding follow-up of HL survivors, the same trend was seen; 66% of GPs reported to provide follow-up care at any level within 5 years since treatment, increasing to 85% for survivors beyond 5 years (Table 1). GPs providing follow-up care to HL survivors both within and beyond 5 years of treatment were more likely to have experience with hospital-based cancer care (*p* = 0.04 and *p* = 0.02, respectively, Table 3). GPs involved in HL follow-up beyond 5 years also reported higher levels of awareness of LEs (*p* = 0.001).

**Figure 1.**
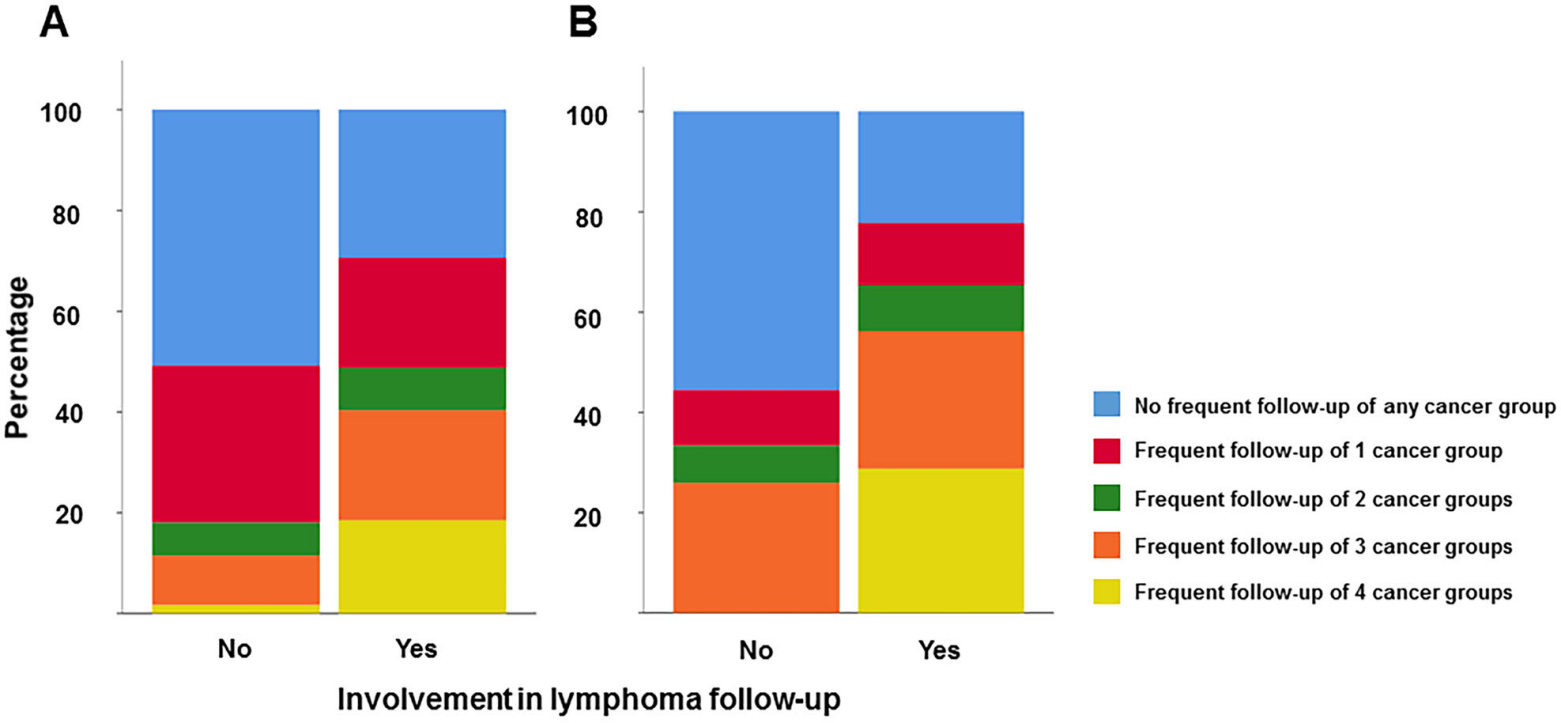
Follow-up care of Hodgkin’s lymphoma versus solid cancers <5 years since treatment (A) and ≥5 years since treatment (B). The stacked bars represent percentages of general practitioners with different levels of involvement in follow-up care of solid cancer diagnoses, *p* < 0.0001 (A) and *p* = 0.011 (B).

GPs engaged in HL follow-up were also more frequently involved in the follow-up of solid tumors, both within and beyond 5 years since treatment, compared to GPs not being involved (*p* < 0.001 and *p* = 0.011 respectively, Figure 1).

### Attitude towards cancer and HL follow-up care and associated factors

When asked about the time point after treatment when the GP would feel comfortable taking responsibility for follow-up care of solid cancer survivors, only 7 GPs reported never feeling comfortable and only 10 being comfortable immediately, with very similar attitudes towards HL follow-up care (Table 1). According to time categories, 38%, 31% and 31% of GPs would feel comfortable taking responsibility for follow-up 0–3 years, >3–5 years and >5 years/never after treatment completion of solid cancers respectively (Table 4). GPs reported similar attitudes towards HL follow-up care with 33%, 28% and 39% being comfortable with follow-up after 0–3, >3–5 and >5 years/never, respectively. The willingness to provide follow-up of survivors of solid cancers sooner after treatment completion was significantly associated with GPs’ age (*p* = 0.05) and experience in general practice (*p* = 0.02) with similar trends for HL survivors (*p* = 0.08 and *p* = 0.05, respectively). The willingness to take responsibility for HL survivors earlier was also associated with familiarity with guidelines on LEs after radiotherapy for lymphoma (*p* = 0.04).

**Table 4.** General practitioners’ preference on time point for taking responsibility for follow-up care.

	Preferred time point for taking responsibility^a^							
	0–3	>3–5 years	>5 years/never	*p* ^c^	0–3	>3–5 years	>5 years/never	*p* ^c^
	Solid cancer				Hodgkin’s lymphoma			
GP, *n* (%)	70 (38)	56 (31)	57 (31)		61 (33)	51 (28)	71 (39)	
Age, years, mean (SD)	50.8 (12.5)	47.0 (12.4)	45.6 (11.8)	**0.05**	50.8 (12.8)	47.6 (12.4)	46.0 (11.7)	0.08
Experience, years, mean (SD)	19.8 (12.9)	15.5 (11.9)	14.1 (11.4)	**0.02**	19.8 (13.2)	15.7 (12.3)	14.8 (11.1)	0.05^c^
Sex, male, *n* (%)	41 (59)	27 (48)	29 (51)	0.48	38 (62)	25 (49)	34 (48)	0.20
Registered specialist, *n* (%)^b^	53 (77)	34 (62)	36 (66)	0.17	44 (73)	33 (66)	46 (67)	0.64
Experience hospital-based cancer care, *n* (%)^b^	22 (31)	23 (41)	17 (30)	0.41	23 (38)	14 (28)	25 (36)	0.49
LE awareness score, mean (SD)	3.6 (1.6)	3.3 (1.2)	3.4 (1.4)	0.56	3.4 (1.7)	3.4 (1.4)	3.4 (1.4)	0.99
Perceived challenges in follow-up care, *n* (%)								
Deficient and/or delayed discharge papers	27 (39)	16 (29)	18 (32)	0.47	23 (38)	15 (29)	23 (32)	0.64
Uncertainty about guidelines	33 (47)	32 (57)	33 (58)	0.39	28 (46)	29 (57)	41 (58)	0.34
Uncertainty about role and division of responsibility	42 (60)	39 (70)	36 (63)	0.53	38 (62)	33 (65)	46 (65)	0.95
Unavailable specialist appointments	18 (26)	12 (21)	19 (33)	0.35	14 (23)	13 (26)	22 (31)	0.57
Perceived responsibility for GPs in follow-up, *n* (%)								
Assist with rehabilitation and disability benefits	56 (80)	44 (79)	46 (81)	0.96	48 (79)	41 (80)	57 (80)	0.97
Detect relapse	61 (87)	53 (95)	51 (90)	0.37	54 (89)	47 (92)	64 (90)	0.81
Provide information on Les	34 (49)	24 (43)	23 (40)	0.63	32 (53)	24 (47)	25 (35)	0.12
Assist with other health issues	56 (80)	36 (64)	41 (72)	0.14	48 (79)	36 (71)	49 (69)	0.43
Opinion on collaboration with hospital, *n* (%)								
Very good/good	46 (66)	31 (55)	16 (28)	**<0.001**	44 (73)	29 (57)	20 (28)	**<0.001**
Acceptable	18 (26)	21 (38)	30 (53)		13 (22)	19 (37)	37 (52)	
Bad/very bad	5 (7)	3 (7)	11 (19)		3 (5)	3 (6)	14 (20)	
Awareness of official guidelines on LEs after cancer treatment in general, *n* (%)	22 (31)	9 (16)	14 (25)	0.14	19 (31)	11 (22)	15 (21)	0.35
Awareness of official guidelines on radiotherapy for lymphoma, *n* (%)	14 (20)	4 (7)	5 (9)	0.06	13 (21)	5 (10)	5 (7)	**0.04**

^a^Percentage based on number of valid responses, missing responses in between 2 and 6.

^b^Experience and specialization concerns general practice.

^c^*p*-Value for Chi-square test for categorical variables and *t*-test/ANOVA for continuous variables. *p*-Values below 0.05 in bold.

GP: general practitioner; LEs: late effects; SD: standard deviation.

When asked about perceived responsibilities in follow-up care, 90% reported surveillance for relapse as the most important role, followed by help with other health issues, rehabilitation, access to social benefits and informing about LEs (Table 1). None of these factors were significantly associated with the time point the GP would feel comfortable with taking responsibility for follow-up (Table 4).

Within the first 5 years since treatment, 87% of GPs reported to collaborate with hospital-based specialists, while 47% reported such collaboration beyond 5 years (Table 1). The perceived quality of such collaboration was significantly associated with the willingness to take responsibility for follow-up early, both for solid cancer and HL survivors (Table 4). The perceived biggest challenges with collaboration were unclear roles and delegation of responsibilities (64%) followed by unclear guidelines for follow-up care (53%) (Table 1).

## Discussion

The GPs’ self-reported level of awareness of LEs was found to be associated with experience, being a specialist in general practice, awareness of guidelines and being female. The level of involvement in follow-up care increased with a higher LE awareness. A small proportion of GPs reported never to feel comfortable with the responsibility of follow-up care. Willingness to take responsibility sooner increased with GP’s age, years of experience and perceived quality of collaboration with hospital specialists. Overall, findings relating to GPs’ involvement and attitude towards follow-up of survivors of HL were similar to survivors after common solid cancers, indicating that challenges in GP-based survivorship care may not be fundamentally different for rare malignancies, such as HL.

The 185 respondents were representative of Norwegian GPs in terms of age, sex and specialist status [17], with a fair response rate of 62%, higher than another similar survey [16]. Still, the sample size may limit generalizability and power to detect differences between groups of respondents. For instance, the estimated effect of guidelines in our statistical analysis may be hampered by the low percentage of GPs that reported being aware of them. The design of our questionnaire is a compromise, with a comprehensive questionnaire more likely returned by GPs with a higher interest in cancer care, thus limiting generalizability. We, therefore chose to focus on a few important somatic LEs with relevance to several cancer diagnoses, not differentiating LEs for individual malignant diseases. This approach limits the possibility to explore the GPs’ in-depth knowledge of the multitude of adverse outcomes after cancer, be it somatic, psychological or social LEs. Due to the way GPs were identified in our study, we expected all to provide long-term follow-up care to HLsurvivors. Still, 15% of the GPs reported not providing follow-up care to HL survivors beyond 5 years after completion of treatment. Possible explanations for this discrepancy may be the limited needs of some long-term HL survivors or the registered GP having been replaced at the time of the survey.

Our results indicate that specialists in general practice and those with experience from hospital-based cancer care had higher levels of awareness of LEs, on average they knew of 0.61 and 0.49 more LEs than non-specialists and those without this kind of experience. As such, education and practice in cancer- and survivorship-care during residency may be of benefit. Educational measures addressing survivorship issues have been desired by residents and specialists in general practice [18,19]. However, although different educational programs on survivorship care are generally felt to be beneficial by GPs, clinical effectiveness is rarely reported [20]. We found that GPs’ familiarity with guidelines on LEs after cancer treatment was associated with increased awareness of LEs. Of concern, less than a quarter of the GPs were aware of the two addressed sets of guidelines. Similarly, in a study of preparedness for survivorship care, Geramita et al. [18] found only 17% of primary care providers to be familiar with relevant American breast cancer guidelines. Reassuringly, however, 61% of the GPs who were familiar with the guidelines used them in practice [18], similar to 74% in our study. Better distribution of guidelines may improve awareness of LEs in survivorship care, in competition however with the abundance of guidelines the GPs have to consider. With the possible multitude of other health issues and comorbidities faced by cancer survivors, implementing guidelines for single diseases may even cause negative consequences for multimorbid patients [21].

In our study, the majority of GPs were involved in follow-up care of solid cancers within the first 5 years since treatment, and involvement increased further beyond this time, similar to previous findings [16]. The degree of collaboration with hospital specialists decreased with time since treatment. These observations may seem to mirror the prevalence of cancer survivors in general and the complexity of their needs by elapsing time after treatment. GPs were less involved in lymphoma follow-up care, most likely due to the lower prevalence of HL survivors. In our study we found awareness of LEs to be positively associated with the degree of involvement in follow-up after treatment for both solid cancer and lymphoma, possibly reflecting GPs’ acquisition of awareness with increasing experience. Alternatively, alert GPs may actively seek and provide survivorship care on a more regular basis. Bober et al. found that GPs with fewer than 10 years of experience were less likely to deliver multidimensional survivorship care than their more experienced peers [22]. In our study, although not reaching statistical significance, GPs with the highest level of involvement tended to be older, more likely specialists, and have longer work experience in general practice, findings indicating the need to support younger professionals.

In our study, GPs show a positive attitude towards taking responsibility for survivorship care beginning early after treatment and increasing with time thereafter. Canadian GPs were commonly involved in follow-up beyond 5 years after diagnosis, but with proper guidelines, were willing to assume responsibility for solid cancer and lymphoma survivors 2.5–3.5 years after the end of treatment [23]. A more positive attitude towards earlier responsibility was reported by older and more experienced GPs in our study. Similarly, other studies have shown experience to be associated with an increased readiness to take responsibility for HL survivors in particular [24] and other aspects of survivorship care in general [25]. Again, these observations suggest strengthening younger and less experienced physicians. In this regard, improved collaboration with hospital specialists may be instrumental. Not only was the perceived quality of such collaboration associated with the attitude towards follow-up, but as pointed out by others, more than half of the GPs in our study reported shortcomings of the interaction with hospitals to be challenging in follow-up care [16,25]. Collaboration with the GPs may be improved, for example, by comprehensive discharge summaries during and after treatment, care plans with a clear division of responsibilities and incorporation of relevant guidelines [23]. Several models on shared follow-up care exist, but little is known about their effectiveness, and no consensus is reached on any preferred model [26,27]. Future GP-based survivorship care plans would however need to incorporate the demands and attitudes of the GPs.

Of GPs in our study, 90% reported detecting relapse as one of the most important tasks, comparable to the 90% and 94% reported by Frew et al. [28] and Greenfield et al. [29], but lower than the around 50% found by Fidjeland et al. [16]. Studies comparing follow-up in primary care versus by hospital specialist after colon [30] and breast cancer [31] did not show differences in recurrence rates, survival or patients’ well-being. In many types of lymphoma, detection of relapse is currently based mostly on patient symptoms and clinical evaluation may be well suited for GP-based surveillance [32]. Empowerment of patients may be an important aspect of GP-based follow-up, educating them about both symptoms of relapse and LEs, with reassurance from readily available caregivers. The use of electronic patient-reported outcomes has been suggested [33], possibly preventing delays in reporting symptoms and leading to earlier detection of relapse [34].

In conclusion, LE awareness, involvement and attitude towards follow-up of cancer survivors in general practice seem to be associated with GPs’ experience from general practice or hospital cancer care, specialization in general practice and awareness of guidelines. GPs’ involvement and attitude did not appear to differ for survivors after common cancer diagnoses and HL, a rare malignancy of young adults.
